# Gene Expression Landscape of Chronic Myeloid Leukemia K562 Cells Overexpressing the Tumor Suppressor Gene PTPRG

**DOI:** 10.3390/ijms23179899

**Published:** 2022-08-31

**Authors:** Giulia Lombardi, Roberta Valeria Latorre, Alessandro Mosca, Diego Calvanese, Luisa Tomasello, Christian Boni, Manuela Ferracin, Massimo Negrini, Nader Al Dewik, Mohamed Yassin, Mohamed A. Ismail, Bruno Carpentieri, Claudio Sorio, Paola Lecca

**Affiliations:** 1 Faculty of Computer Science, Free University of Bozen-Bolzano, Piazza Domenicani 3, 39100 Bolzano, Italy; 2 Department of Medicine, Division of General Pathology, University of Verona, Strada Le Grazie 8, 37134 Verona, Italy; 3 Department of Cancer Biology and Genetics, Comprehensive Cancer Center, The Ohio State University, Columbus, OH 43210, USA; 4 Department of Experimental, Diagnostic and Specialty Medicine, University of Bologna, Via S. Giacomo 14, 40126 Bologna, Italy; 5 Dipartimento di Medicina Traslazionale e per la Romagna, University of Ferrara Via Fossato di Mortara 70, 44121 Ferrara, Italy; 6 Department of Pediatrics and Neonatology, Neonatal Intensive Care Unit, Newborn Screening Unit, Women’s Wellness and Research Center, Hamad Medical Corporation, Doha 3050, Qatar; 7 Genomics and Precision Medicine (GPM), College of Health & Life Science (CHLS), Hamad Bin Khalifa University (HBKU), Doha 34110, Qatar; 8 Department of Research, Women’s Wellness and Research Center, Hamad Medical Corporation, Doha 3050, Qatar; 9 Interim Translational Research Institute (iTRI), Hamad Medical Corporation (HMC), Doha 3050, Qatar; 10 School of Life Science, Pharmacy and Chemistry, Faculty of Science, Engineering & Computing, Kingston University, Kingston University London, River House, 53–57 High Street, Kingston upon Thames, London KT1 1LQ, UK

**Keywords:** differential expression gene analysis, enrichment gene analysis, bio-ontologies, Chronic Myeloid Leukemia

## Abstract

This study concerns the analysis of the modulation of Chronic Myeloid Leukemia (CML) cell model K562 transcriptome following transfection with the tumor suppressor gene encoding for Protein Tyrosine Phosphatase Receptor Type G (PTPRG) and treatment with the tyrosine kinase inhibitor (TKI) Imatinib. Specifically, we aimed at identifying genes whose level of expression is altered by PTPRG modulation and Imatinib concentration. Statistical tests as differential expression analysis (DEA) supported by gene set enrichment analysis (GSEA) and modern methods of ontological term analysis are presented along with some results of current interest for forthcoming experimental research in the field of the transcriptomic landscape of CML. In particular, we present two methods that differ in the order of the analysis steps. After a gene selection based on fold-change value thresholding, we applied statistical tests to select differentially expressed genes. Therefore, we applied two different methods on the set of differentially expressed genes. With the first method (Method 1), we implemented GSEA, followed by the identification of transcription factors. With the second method (Method 2), we first selected the transcription factors from the set of differentially expressed genes and implemented GSEA on this set. Method 1 is a standard method commonly used in this type of analysis, while Method 2 is unconventional and is motivated by the intention to identify transcription factors more specifically involved in biological processes relevant to the CML condition. Both methods have been equipped in ontological knowledge mining and word cloud analysis, as elements of novelty in our analytical procedure. Data analysis identified RARG and CD36 as a potential PTPRG up-regulated genes, suggesting a possible induction of cell differentiation toward an erithromyeloid phenotype. The prediction was confirmed at the mRNA and protein level, further validating the approach and identifying a new molecular mechanism of tumor suppression governed by PTPRG in a CML context.

## 1. Introduction

Chronic Myeloid Leukemia (CML) is a myeloproliferative disease affecting approximately 1 per 200,000 persons per year in industrialized countries. Many treatment improvements have been achieved recently, especially in the development of new drugs, but a mortality rate of 2–3% per year remains [1,2]. A distinctive feature of CML is the reciprocal translocation, originating in hematopoietic stem cells (HSCs), between the long arms of chromosomes 9 and 22, i.e., t(9;22) (q34;q11.2), which results in the BCR-ABL1 chimeric gene. This genomic aberration generates a new fusion gene, BCR-ABL1, which encodes for a tyrosine kinase held accountable for the neoplastic transformation of these cells by affecting normal cellular pathways essential for tissue homeostasis and thus causing the alteration of crucial cellular processes, such as apoptosis, cell cycle, and autophagy [3,4]. In this context, one primary goal of research is to identify the regulatory mechanisms antagonizing the kinase activity of BCR-ABL1 and, possibly, of other vital effectors intersecting this pathway, as players other than BCR-ABL1 have been involved in the pathogenesis of the disease [5,6]. The natural history of CML, prior to the advent of small molecule protein kinase antagonists, features a progression from a stable or chronic phase to an accelerated phase or to a rapidly fatal blast crisis within 3–5 years. Typically blood cells differentiate normally in the stable phase, but not in the blast phase [1]. Protein Tyrosine Phosphatase Receptor Type G (PTPRG) is a member of the protein tyrosine phosphatase (PTP) family featuring an extracellular and a single transmembrane region and two tandem intracytoplasmic catalytic domains [7]. PTPRG is widely expressed in human tissues [8] and is involved in the regulation of cell growth, differentiation, mitotic cycle, and oncogenic transformation [9,10]. The gene encoding for this phosphatase is located in a chromosomal region (3p21-p14.2) frequently deleted in renal cell and lung carcinoma, where PTPRG acts as a tumor suppressor in many cancers  [11,12,13,14]. Specifically, PTPRG was recognized as having an oncosuppressor function gene and was found down-regulated in CML patients. The relevance of this gene to CML has recently been supported by several studies performed in patients, and strategies aimed at restoring its expression are expected to benefit the course of the disease by improving drug efficacy or contrasting the emergence of BCR/ABL1 mutants [15,16,17].

Epigenetic events, such as the hyper-methylation of its promoter region as well as intron 1, negatively regulate the transcription of PTPRG, as demonstrated in CML and childhood acute lymphoblastic leukemia [16,18,19,20]. Re-expression of this protein occurs in leukocytes (especially neutrophils) of CML patients following targeted therapy [18]. Once activated, PTPRG can reduce the phosphorylation level of BCR-ABL1 and some of its key targets, such as CRK-L and STAT5 [18]. We found that in CML cells, PTPRG expression inversely correlates with BCR/ABL1 expression and activation, both in cell lines and primary cell models following pathways that include beta catenin [21] and possibly others that are currently under investigation [17,20,21].

Our study focuses on the detection of genes and gene pathways in protein–protein interaction networks (commonly considered a proxy of gene networks) that are most likely affected by the state of the gene coding for PTPRG and by the treatment with a prototype tyrosine kinases inhibitor (TKI), Imatinib, in the K562 cell line overexpressing the enzymatic active and enzymatic dead PTPRG. Tyrosine kinases phosphorylate proteins on tyrosine residues, producing a biologic signal that also influences many aspects of cellular functions, including cell growth, proliferation, differentiation, and death. PTPs act as natural modulators of TKI signaling, and it is well known how the inhibition of TKI represents a strategy to disrupt signaling pathways that promote neoplastic growth and survival in haematologic malignancies and likely in other neoplasia as well. In order to identify responsive genes, we implemented two analytical pipelines, hereafter referred to as Method 1 and Method 2. On the set of differentially expressed genes, we applied two methods of analysis. With the first method (Method 1), we implemented Gene Set Enrichment Analysis (GSEA [22]), followed by the identification of transcription factors. With the second method (Method 2), we first selected the transcription factors (TFs) from the set of differentially expressed genes and implemented GSEA on this set. Method 1 is a standard method commonly used in this type of analysis, while Method 2 is unconventional and is motivated by the intention to identify transcription factors more specifically involved in biological processes relevant to the CML condition. In Method 1, due to a larger gene universe, we expect the set of transcription factors selected upstream of the GSEA to be either larger or related to the known role of PTPRG as a modulator of hematopoietic cell differentiation [10].

## 2. Materials and Methods

In this section, we report on the methods and materials relevant to the experimental activity of data collection and the methodology of the computational analysis of the data.

### 2.1. Cell Lines

The human K562 Chronic Myeloid Leukemia clones expressing full-length PTPRG, empty vector, and inactive mutant holding a mutation on the catalytic domain D1028A were previously described [18] and were cultured in RPMI medium supplemented with 1% L-glutamine 100× (Biowest), 10% fetal bovine serum (FBS, Euroclone), and the selective agent G418 0.5 mg/mL (Sigma) at 37${}^{\circ }$ C in a humidified incubator with 5% CO${}_{2}$.

### 2.2. Quantitative Real-Time Polymerase Chain Reaction

Total RNA was extracted from the K562 cell lines using Qiagen RNeasy Kit according to the manufacturer’s protocol. Complementary DNA was synthesized using the PrimeScript™ reagent Kit (TAKARA BIO Inc, Shiga, Japan), and the quantitative real-time polymerase chain reaction (qRT-PCR) was performed using TB Green Premix™ Ex taq (TAKARA BIO Inc.). Each sample was run in triplicate, and 3 ng complementary DNA was used for each reaction. The sequences of gene-specific primers used are listed in Table 1. The fold changes in mRNA levels of transcription factors (TF-DEGs) between K562 cell line expressing PTPRG and control group were determined using the 2${}^{-\Delta \Delta \mathrm{Ct}}$ method with GAPDH used as the internal control for normalization. Prism GraphPad Software [23]) was used for statistical analyses, and the Student’s t-test was used to determine statistically significant differences between groups.

### 2.3. Flow Cytometry Analysis

The K562 cell lines (5 × 105 cells) were harvested, washed in FACS buffer (PBS supplemented with 2% FBS and 2 mM EDTA), and centrifuged at 1200 rpm for 5 min at room temperature. The cell suspensions (100 μL) were plated in 96-well plate, and 2 μL anti-CD36 (V450 mouse 2-Human CD36; cat.no. 561535; BD Biosciences) was added. The samples were incubated in the dark for 1 h at 4 ${}^{\circ }$C, washed again with FACS buffer, and centrifugated (1200 rpm for 5 min). FACS buffer (150 μL) was added to the cell pellet, and the samples were analyzed using MACSQuant Analyzer 10 Flow Cytometer (Miltenyi Biotec) [24]. The data were analyzed with FlowJo^TM^ v10.8.1 software [25], and the fraction of positively stained cells (CD36+) was determined as the percentage of live population stained with Propidium Iodide (PI).

### 2.4. Data Collection

The RNAs from the samples were hybridized on Agilent whole human genome oligo microarray (#G4851A, Agilent Technologies, Palo Alto, CA, USA). This microarray consists of 60 mer DNA probes synthesized with SurePrint technology [26], covering 60,000 unique human transcripts. One-colour gene expression was performed according to the manufacturer’s procedure. Briefly, total RNA fraction was obtained from samples by using the Trizol Reagent (Invitrogen). RNA quality was assessed by the use of Agilent 2100 Bioanalyzer (Agilent Technologies). Low quality RNAs (RNA integrity number below 7) were excluded from microarray analyses. Labeled cRNA was synthesized from 100 ng of total RNA using the Low Input Quick-Amp Labeling Kit, one colour (Agilent Technologies) in the presence of cyanine 3-CTP. Hybridizations were performed at 65 ${}^{\circ }$C for 17 h in a rotating oven. Images at 3 μm resolution were generated by Agilent scanner, and the Feature Extraction 10.7.3.1 software (Agilent Technologies) was used to obtain the microarray raw data.

Microarray results were then analysed by using the GeneSpring GX 11 software (Agilent Technologies). Data transformation was applied to set all the negative raw values at 1.0, followed by a normalization on the 75th percentile. A filter on low gene expression was used to keep only the probes expressed in at least one sample (flagged as Marginal or Present).

The data used in this study derive from the above-mentioned analysis carried out by microarray hybridization of the CML cell transcriptome (K562) in different conditions. The cells were transfected with full-length PTPRG and compared to several controls: cells transfected with the empty vector, cells transfected with PTPRG inactive mutant holding a mutation on the catalytic domain (D1028A), and cells treated with Imatinib targeting the oncogene BCR/ABL1. We integrated the data relating to gene expression with the gene ontology and protein–protein network data. We investigated

- The effect of the PTPRG expression and its activation status;
- The impact of PTPRG expression (both active and inactive) in the presence of TKI, hereafter called by its clinical name Imatinib;
- The effect of Imatinib in combination with functional or mutant PTPRG expression.

For this purposes, we developed ad hoc methods to identify differentially expressed genes, with a particular focus on gene coding for transcription factors. This class of genes was selected, as they are known to act as master genes activating cell programs that include key features, such as cell differentiation and proliferation, rather than controlling genes essential for the ontogenesis and maintenance of the normal hematopoietic system, and instead of being involved in the pathogenesis of leukemia [27].

### 2.5. Computational Analysis

We implemented first differential gene expression analysis and then gene ontology enrichment analysis of the identified differentially expressed genes (DEGs). Differential expression analysis (DEA) is a single-gene technique performed to identify differentially expressed genes (DEGs), namely genes whose expression levels vary significantly under different experimental conditions. Gene set enrichment analysis (GSEA) is a computational method applied to get biological insights from gene expression data. It is typically used to examine a given subset of interesting genes stemming from previous analyses versus an extensive reference set referred to as gene universe. Unlike single-gene techniques, GSEA aims at identifying statistically significant groups of functionally related genes by relying on current knowledge for data classification. For theoretical in-depth analyses of GSEA, refer to [22]. Depending on the specific biological question that is designed to be tackled, several databases can be employed to investigate a priori gene functional groupings.

#### 2.5.1. Differential Gene Expression Analysis

In this study, differential gene expression analysis was performed to detect DEGs between two groups: control and the phosphatase inactive mutant D1028A [18] samples considered to be the untreated group (4 replicates) and the treatment group referred to as PTPRG-expressing samples (2 replicates). Differential expression analysis was conducted on log2-transformed data using the Bioconductor/R package limma (Version 3.12) [28]. Both the empirical Bayes correction on the variances and the multi-testing Benjamini–Hochberg correction on *p*-values were selected.

Among the set of differentially expressed genes, we focused on transcription factors (TF-DEGs). Indeed, the identification of the TF-DEGs responsive to the treatments would allow for identifying the active drivers turning specific genes (possibly involved in the onset and progression of CML) “on” or “off” or boosting/repressing the gene’s transcriptions.

#### 2.5.2. Gene Ontology Enrichment Analysis of DEGs

In this study, gene ontology enrichment analysis of the DEGs relied on the Gene Ontology (GO) system of classification [29] and in particular on the GO domain referring to biological processes. Therefore, over-representation of GO terms pertinent to the DEGs previously identified has been tested to reveal associations with disease phenotypes. Instead of investigating the results of GSEA applied to the whole set of DEGs, we focused on the transcription factors (TFs) detected as DEGs (TF-DEGs).

The analysis of transcription factors was carried out by applying two different GSEA methods implemented by the Bioconductor/R package topGO (version 3.12) [30]. Both methods combine a classical enrichment analysis with the Kolmogorov–Smirnov statistic test (runTest function with input parameters “algorithm = classic” and “statistic = ks”). This particular setting was selected for two reasons.

1. The methods compute the significance of a node independently from its neighboring nodes [31]. This means that if a GO term contains the same genes as one of its children, then the traditional method gives the children the same score. While this setting could cause data redundancy, by not discarding any GO term based on parent–child relationships, it allows for keeping valuable information that can be exploited later on to investigate associations and dependencies between GO terms.
2. The Kolmogorov–Smirnov statistic computes enrichment based on gene scores [29]. Hence, it is possible to take full advantage of the information obtained by DEA by ranking genes according to their adjusted *p*-values.

**Figure 1:**
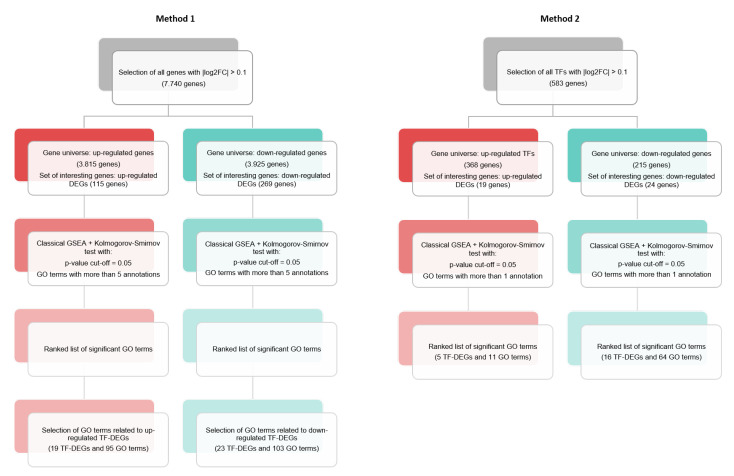
GSEA-based methods.

These two considerations lead to two methods, hereafter referred to as Method 1 (corresponding to consideration 1) and Method 2 (corresponding to consideration 2), which are outlined in more detail in Figure 1. Both methods were developed on two separate streams to discriminate between GO terms associated with up-regulated and down-regulated genes, respectively. In this regard, the procedure returned a total of four lists of significant GO terms split into two methods and subsequently into two modalities (up-regulated and down-regulated). The output lists of significant GO terms obtained were analyzed on a textual content level and compared modality-wise with the aim of extracting text-based insights that could guide the reader in searching GO terms relevant to the case study and discriminating between the two methods at a glance. In these regards, a graphical technique was developed based on word clouds for visual representation and GSEA rankings for computing single-word significance. Specifically, the technique was based on the R packages wordcloud [32] and tm [33].

Its main steps are outlined in the following.

1. **GSEA**
  - Ranked list of significant GO terms.
2. **Data cleaning**
  - The text is converted to lower case.
  - Unnecessary white spaces, common stop words, and punctuation are removed from the text.
3. **Term document matrix**
  - Each GO term is chunked into a single word.
  - Each word is ranked based on the *p*-value of the GO term it belongs to. The rank is called *order*.
  - Word significance for a given chunk is defined as the sum of the reciprocals of the respective orders retrieved by running down the whole list.
  - Repeated chunks are removed, and the final list, i.e., the term document matrix, is sorted based on word significance.
4. **Visual representation**
  - The word cloud is displayed.
  - Correlation analysis between chunks is performed for the first top 10 words of the list.
  - The results are shown in a bubble plot.

Tasks 1–4 have been applied to all the GO lists returned by Method 1 and Method 2.  

The word clouds and the correlation plots were used to inspect the lists of significant GO terms returned by the two methods, compare the different results, and carry out in-depth analyses on a specific subset of labels. In this regard, significant GO terms—and hence biological processes—plausibly correlated with CML have been selected and further examined at a single-gene level to implement the following objectives.

1. To compare the informative content of the labels to optimize the identification of genes relevant to CML. More generic and high-level labels were discarded in favor of more CML-specific ones.
2. To extend the analysis from TF-DEGs to their partners in the gene networks to gain biological insights on gene–gene interactions and better understand the impact of the treatment on the network topology.

## 3. Results

**Figure 2:**
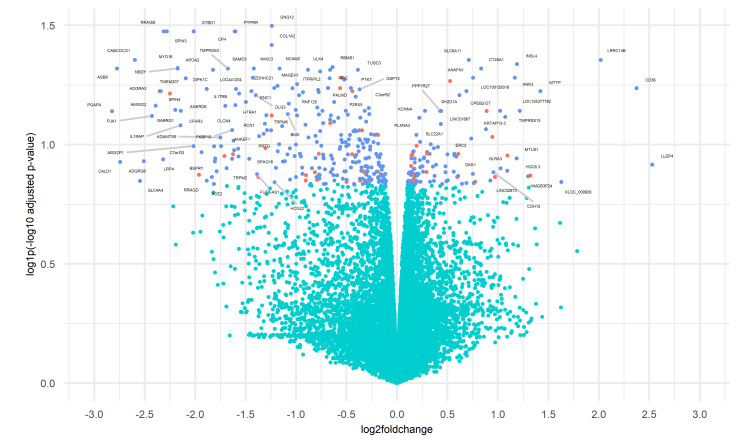
Volcano plot plot for DEGs among controls and PTPRG overexpressing K562. Genes are represented as scattered points: the *x*-axis is the log2FoldChange, and the y-axis shows the log1p(-log10 adjusted *p*-value). Green dots represent the non-differentially expressed genes. Both red and blue dots represent genes that were identified as significantly differentially expressed (adjusted *p*-value <0.05) with |log2FoldChange|>0.1. Specifically, red dots refer to transcription factors.

Genes with an adjusted *p*-value <0.05 and |log2FoldChange| > 0.1 were considered to be differentially expressed. Based on these criteria, 384 genes were selected as DEGs: 115 were up-regulated and 269 were down-regulated (see Figure 2). In the set of genes scoring positively on the fold change test, we identified 43 differentially expressed transcription factors (TF-DEGs), of which 24 were down-regulated and 19 were up-regulated (see Figure 1). The top five down-regulated TF-DEGs were ARNTL2, ZNF563, KLF7, TRPS1, and LHX2, while the top five up-regulated were ZNF90, ZNF492, HOXD9, MECP2, and RARG.

### Validation

**Figure 3:**
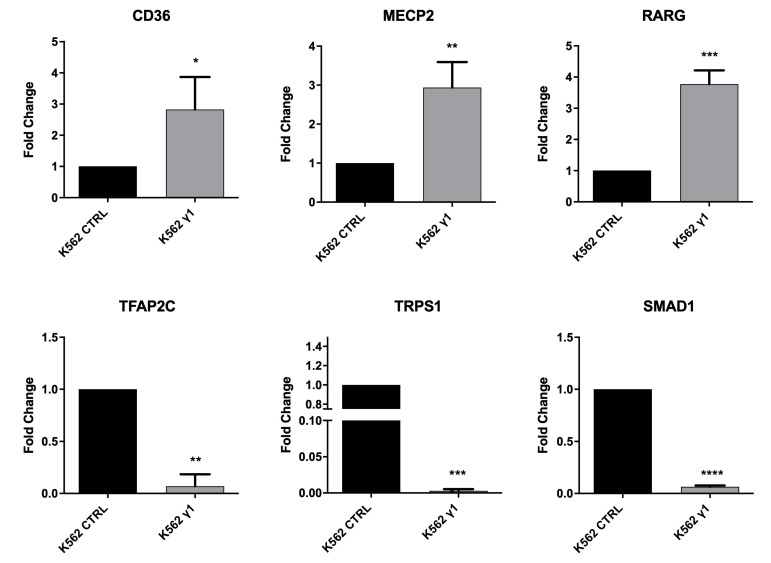
mRNA level of selected up-regulated and down-regulated DEGs identified by Method 1 and 2. The mRNA levels of genes were determined by qRT-PCR, and the relative fold changes were calculated between K562 expressing PTPRG and untreated control group (control ∅ and D1028A). GAPDH was used as the endogenous control. The number of asterisks denotes the order of magnitude of the *p*-value: *p*-values less than 0.0001 are summarized with four asterisks, *p*-values less than 0.001 are summarized with three asterisks, *p*-values less than 0.01 are summarized with two asterisks, and *p*-values less than 0.1 are summarized with one asterisk.

We proceeded to the validation of gene expression by quantitative RT-PCR on an independent set of samples. We selected a group of up- and down-regulated genes based on microarray data and performed RT-qPCR validation. Figure 3 shows the results of the analysis, providing confirmation of the microarray analysis.

**Figure 4:**
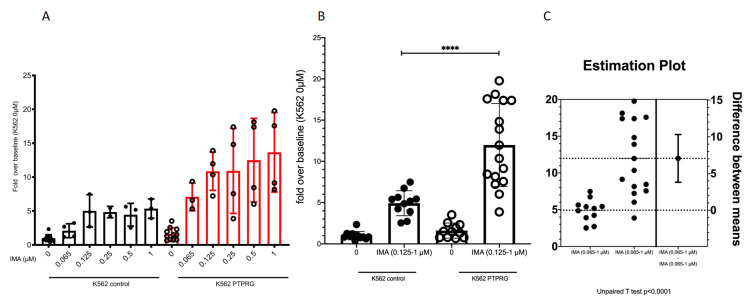
Up-regulation of RARG implies an increased propensity to erythroid differentiation in hemopoietic cells. (**A**) K562 cells were treated for 48 h with the indicated concentrations of Imatinib (IMA). (**B**,**C**) Cells were lysed, and Hb content was evaluated (ng Hbμg total lysate) and expressed as fold increase over baseline (*p*-value < 0.0001 Mann–Whitney test). In (**B**), by convention, the number of asterisks denotes the order of magnitude of the *p*-value: *p*-values less than 0.0001 are summarized with four asterisks.

**Figure 5:**
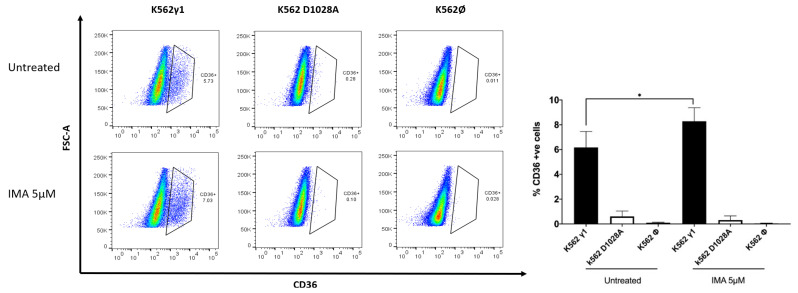
Flow cytometry analysis of CD36+ surface marker on K562 subclones. (**Left panel**): Representative dot plot graphs show the increased expression of CD36 on the surface of the K562 cell lines expressing PTPRG γ1 compared to the control group (control *∅* and D1028A) in presence or absence of 5 μM IMA overnight treatment. (**Right panel**): summary of the results of a minimum of 3 experiments (*p* = 0.03, one-tail *t*-test). *p*-values less than 0.1 are summarized with one asterisk.

DEA and GSEA performed on the TF-DEGs bring to our attention a set of up- and down-regulated genes that become part of a complex network reflecting on cell phenotype. Transcription factors recognize and bind to consensus sequence elements that are specific for each transcription factor, and the transcription factors then regulate downstream gene expression. We then proceeded to evaluate the phenotypic consequences of this regulation and focused on the up-regulation of RARG, a gene belonging to the nuclear receptor superfamily, sharing 90% homology with retinoic acid receptor α (RARα, also indicated as RARG) and retinoic acid receptor β (RARβ), which appears crucial for haematopoietic development [34] and the erythroid differentiation program. Although the effect in mice appears to be the result of an erythroid cell extrinsic role (i.e., alteration of the bone marrow microenvironment), a role in stress erythropoiesis or non-homeostatic erythroid demand was not excluded [34,35]. Therefore, up-regulation of RARG might imply an increased propensity to erythroid differentiation in haemopoietic cells, a prediction that we verified and confirmed in the same cell model. As we noticed that starting from 0.125 μM IMA, the cells seem to have reached the maximum capability to produce haemoglobin, we decided to pool these data and perform statistical analyses, such as an estimation plot (Figure 4). The statistical analyses confirmed the change in the differentiation program toward the erythroid lineage. Furthermore, CD36, expressed by committed erythroid progenitors expressing higher levels of β-globulin [36], appears to be one of the genes more strongly up-regulated, further suggesting that erythroid differentiation is modulated by PTPRG expression. Thus, ultimately, in addition to confirming the up-regulation of CD36 at the mRNA level (as shown previously in Figure 3), we also confirmed it at the protein level both under resting conditions and after overnight treatment with IMA 5 upmuM (Figure 5).

In order to further validate the cDNA assays, we identified four additional genes (STAT1, NFκB p50, C/EBP β, and NFκB p65) that were expressed (but not modulated by PTPRG overexpression). As expected, the genes were also expressed at similar levels at the protein level, further supporting the value of the cDNA array analysis (Figure S1 in Supplementary Materials).

## 4. Identification of Molecular Pathways

**Figure 6:**
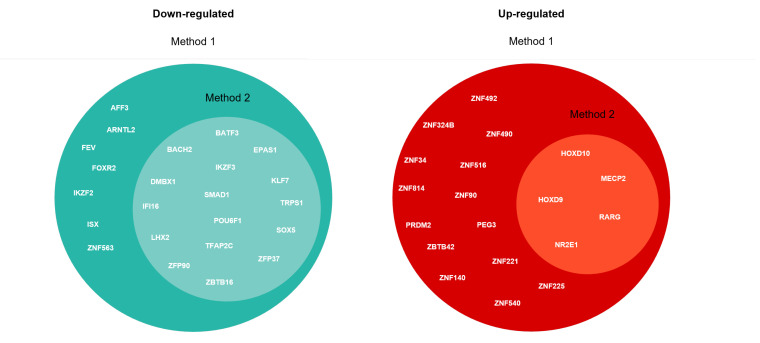
TF-DEGs detected by Methods 1 and 2.

**Figure 7:**
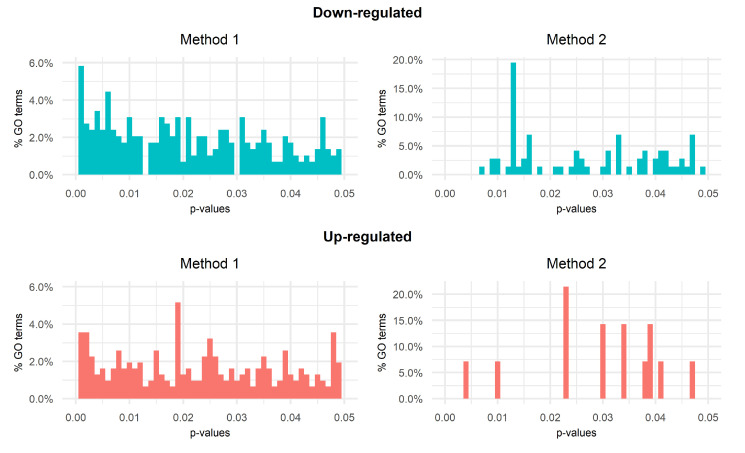
Bar plots of GSEA *p*-values.

As a result of GSEA, the TF-DEGs identified by Methods 1 and 2 are represented in Figure 6. The TF-DEGs associated with GO terms stemming from Method 2 are contained in the set returned by Method 1. Figure 7 shows the bar plots of the distributions of *p*-values returned by Methods 1 and 2. The plots show significant differences between the two methods: Method 1 returns larger sets of GO terms with low *p*-value frequencies (less than 6% for both up-regulated and down-regulated genes). On the other hand, Method 2 returns smaller sets of GO terms characterized by frequencies that reach up to 20%. In this regard, as opposed to Method 1, Method 2 shows fewer significant GO terms distributed in more densely populated and separated clusters.

### 4.1. Automated GSEA-Based Semantic Analysis

**Figure 8:**
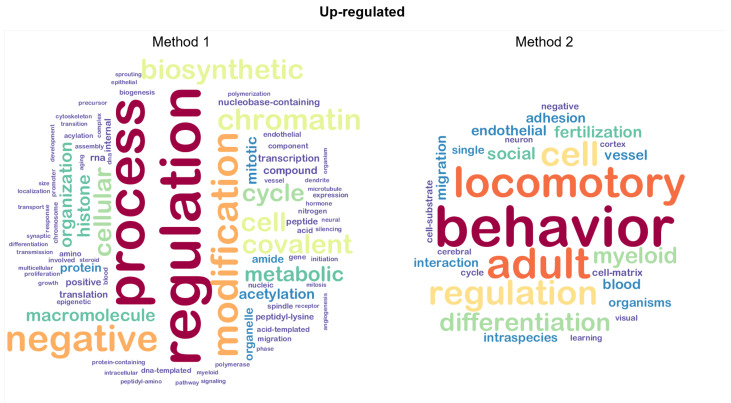
Word clouds for GO terms related to up-regulated genes. The word clouds show the top 100 words retrieved using the procedure described in Figure 7.

In order to better inspect the differences between the two methods, we built and analyzed the weighted word clouds from such lists of GO terms with a view of improving understanding about the differences between Method 1 and Method 2. Word clouds for up-regulated genes are shown in Figure 8. We note a clear distinction between the two plots based on both data quantity and content. For example, the chunk myeloid is contained in both word clouds in two different sizes:

- In the first case, it appears as a small-sized chunk among other terms that are in all likelihood connected to CML;
- In the second case, it is represented as a middle-sized chunk among terms that seem quite distant from the target.

**Figure 9:**
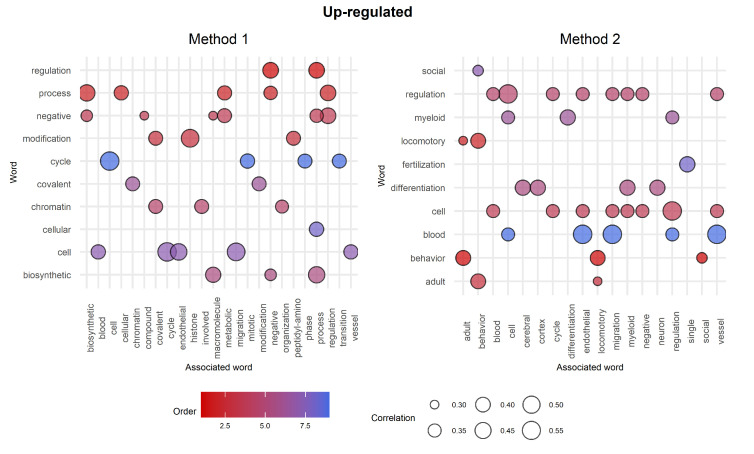
Correlation analysis for the top 10 words. The top 10 most significant words are represented on the y-axis, while their respective associated words are on the *x*-axis. The colour of the bubbles is based on the order (significance of a given GO term as described in Figure 7), whilst the size depends on the correlation between words. Note that if two top 10 words A and B are associated with each other, then the plot shows both pairs (A,B) and (B,A).

Correlation analysis was then performed further to investigate the informative content of the word clouds. Figure 9 shows the results achieved on the top 10 most significant words. The chunk myeloid appears in Method 2’s top 10 associated with the chunks regulation, differentiation, and cell, which in turn show other interesting associations. On the other hand, Method 1 shows interesting correlations for all the top 10 chunks even if the word myeloid is not among them. In this regard, further investigation was conducted by going through the list of GO terms and picking attractive labels based on the insights extracted from the word clouds.

**Figure 10:**
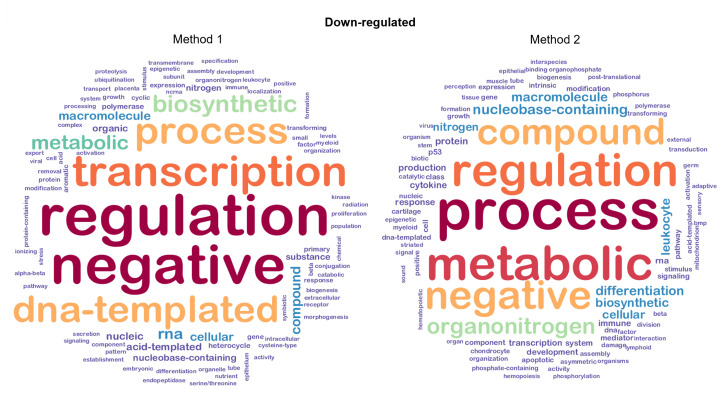
Word clouds for GO terms related to down-regulated genes. The word clouds show the top 100 words retrieved using the procedure described in items 1–4 in Section 2.5.2. Both single word size and colour depend on word significance.

Figure 10 shows the word clouds for down-regulated genes. In this case, the two plots show mainly content-based differences. In fact, both clouds are thick and almost equally distributed in terms of word sizes. Moreover, the most powerful words are mostly in common. Both clouds show words of potential interest for experimental analyses—even if with different sizes—such as immune, transcription, myeloid, leukocyte, and growth. On the other hand, Method 2 appears to be more detailed than Method 1 since it shows additional specific chunks, such as apoptotic, hemopoiesis, hematopoietic, p53, chondrocyte, cytokine, stem, and differentiation.

**Figure 11:**
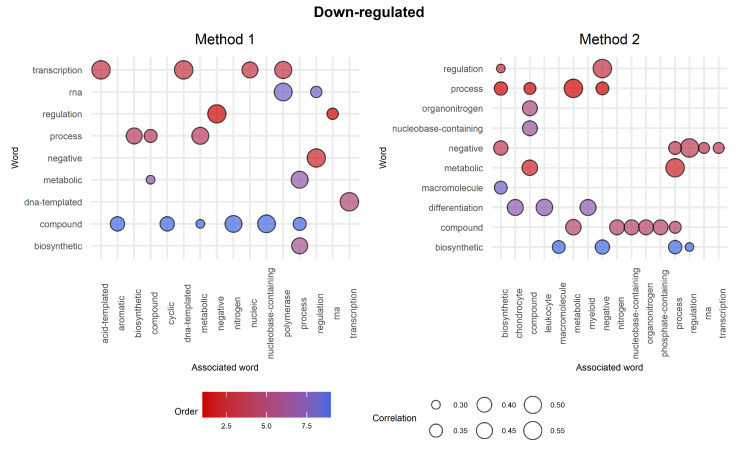
Correlation analysis for the top 10 words. The top 10 most significant words are represented on the *y*-axis, and their respective associated words are on the *x*-axis. The colour of the bubbles is based on the order (significance of a given GO term as described in items 1–4 in Section 2.5.2), whilst the size depends on the correlation between words. Note that if two top 10 words A and B are associated with each other, then the plot shows both pairs (A,B) and (B,A).

Correlation analysis was then performed to better discriminate between the two word clouds. Figure 11 shows the results performed on the top 10 most significant words. We see that the majority of the top 10 words are shared between the two methods. The chunks regulation, process, negative, metabolic, compound, and biosynthetic are represented in both plots. Moreover, since we are analysing biological processes related to down-regulated genes, it is interesting that both methods share the association negative–regulation. However, the main difference between the two methods relies on the associated words rather than on the most significant ones themselves. In fact, Method 1 shows interesting but high-level associations that bring attention to generic biological processes. On the contrary, Method 2 shows more detailed associations, such as differentiation–chondrocyte, leukocyte, myeloid, compound–phosphate-containing, and negative–transcription.

### 4.2. Final Expert-Curated GO Terms Selection

After examining the GO lists on a single-word level with the aim of highlighting key words and biological insights, we thereby selected specific GO terms which showed particular relevance to biological processes involved in CML onset and development. In this regard, the plots presented hereafter split the analysis into two levels, which are the level defined by the GO terms set that provides labels along with the enrichment score (transformed *p*-value) returned by GSEA and (ii) the level of the TF-DEGs set.

**Figure 12:**
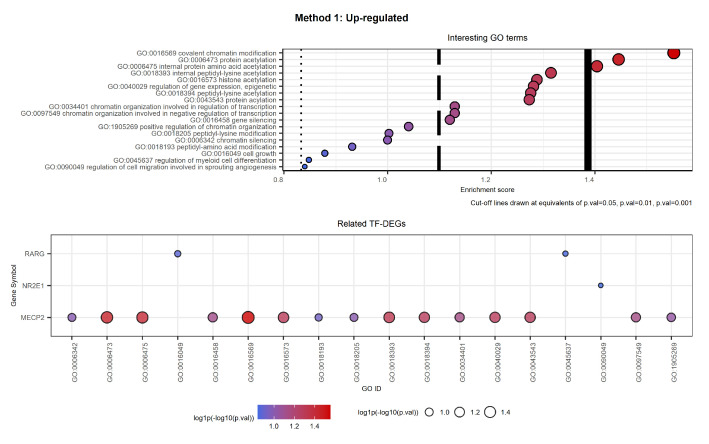
Interesting GO terms and related TF-DEGs. The first plot shows selected GO terms on the *y*-axis and the respective log1p(−log10(*p*-value)) as the Enrichment score on the *x*-axis. The second plot shows on the *y*-axis the TF-DEGs and the related GO terms on the *x*-axis. In both plots, colour and size of the bubbles depend on the Enrichment score. We note that gene MECP2 is involved in almost all the selected GO labels, while genes RARG and NR2E1 result in being specific.

**Figure 13:**
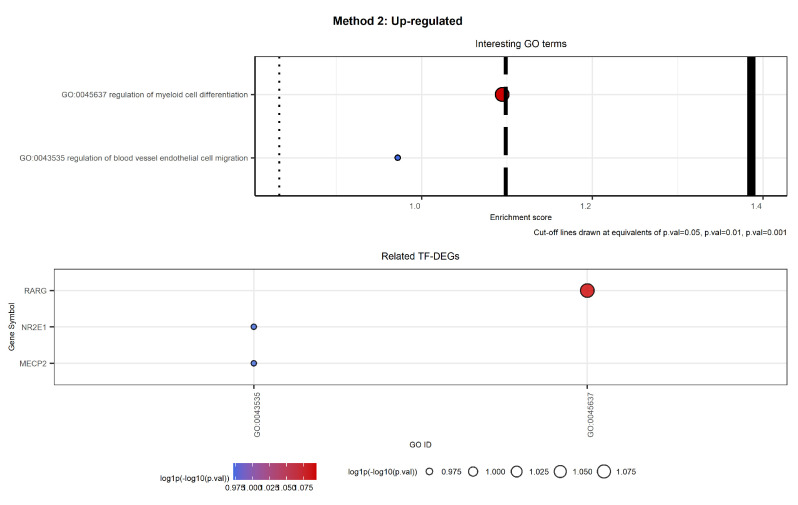
Interesting GO terms and related TF-DEGs. The first plot shows selected GO terms on the *y*-axis and the respective log1p(−log10(*p*-value)) as the Enrichment score on the *x*-axis. The second plot shows TF-DEGs on the *y*-axis and the related GO terms on the *x*-axis. In both plots, colour and size of the bubbles depend on the Enrichment score. We note that gene RARG results in being strictly CML-related, while genes MECP2 and NR2E1 are associated with a more general biological process.

Figure 12 and Figure 13 show the selected labels for up-regulated genes. The first plot of Figure 12 shows the selected labels returned by Method 1. GO terms result in being clustered as in Table 2.

The second plot of Figure 12 shows the TF-DEGs associated with the above-mentioned GO terms. We note that there are only three TF-DEGs: MECP2, involved in almost all the selected biological processes; NR2E1, associated with the regulation of cell migration involved in sprouting angiogenesis; and RARG2, implicated in both cell growth and the regulation of myeloid cell differentiation.

The first plot of Figure 13 shows the selected labels returned by Method 2. There are only two GO labels that show a connection with CML (see Table 3).

Even if the selection is very different from the one carried out in Method 1, we obtained that the TF-DEGs identified are the same. Furthermore, Method 2 detected both NR2E1 and MECP2 as members of GO:0043535.

**Figure 14:**
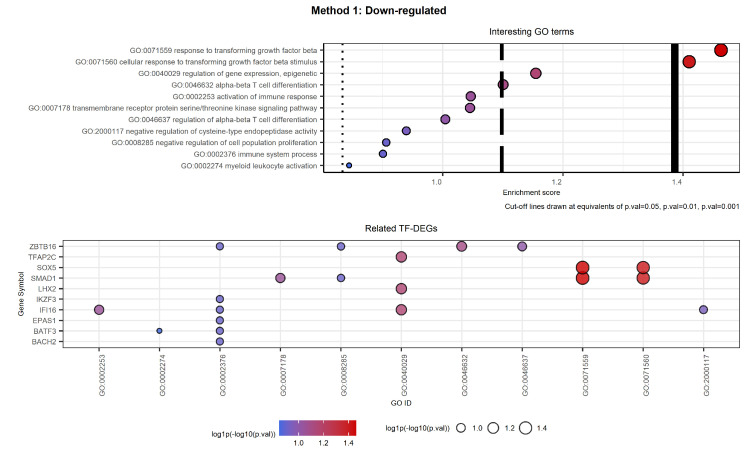
Selected GO terms and related TF-DEGs. The first plot shows selected GO terms on the *y*-axis and the respective log1p(−log10(*p*-value)) as the Enrichment score on the *x*-axis. The second plot shows TF-DEGs on the *y*-axis and the related GO terms on the *x*-axis. In both plots, colour and size of the bubbles depend on the Enrichment score. We note that genes SOX5 and SMAD1 are associated with the most significant GO labels, which results in being associated with cell growth.

**Figure 15:**
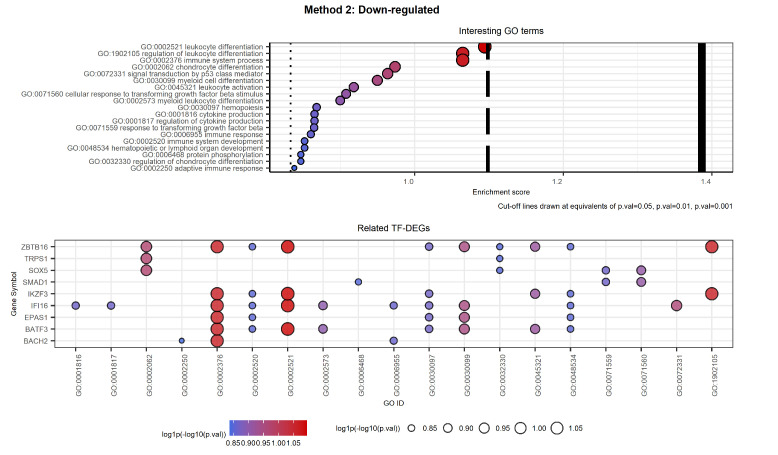
Selected GO terms and related TF-DEGs. The first plot shows selected GO terms on the *y*-axis and the respective log1p(−log10(*p*-value)) as the Enrichment score on the *x*-axis. The second plot shows TF-DEGs on the *y*-axis and the related GO terms on the *x*-axis. In both plots, colour and size of the bubbles depend on the Enrichment score. We note that both genes MECP2 and NR2E1 are involved in the same process, while RARG is identified as strictly CML-related. We note that almost all genes are associated with the most significant GO labels involving leukocyte differentiation.

Figure 14 and Figure 15 show the selected labels for down-regulated genes. The first plot of Figure 14 shows the selected labels returned by Method 1. GO terms can be clustered as follows based on the key words used to carry out the selection reported in Table 4.

The second plot shows the TF-DEGs associated with the GO terms listed in Table 4. In the plot, we see that the two most significant GO terms both refer to the genes SOX5 and SMAD1. Moreover, GO:0071560 is a direct child of GO:0071559, and hence it is more specific than the other. Secondly, we note that several genes are identified in the high-level label GO:0002376 immune system process. Among them, only ZBTB16, IFI16, and BATF3 are associated with more specific terms.

The first plot of Figure 15 shows the selected labels returned by Method 2. The selection of GO terms reported in Table 5 is more relevant to CML than the one returned by Method 1, both regarding the number of labels and their specificity. On the other hand, the set of related TF-DEGs overlaps with the one from Method 1 except for the genes LHX2 (only in Method 2), TFAP2C, and TRPS1 (only in Method 1). Moreover, it is possible to notice that the TF-DEGs are always associated with more than two labels. Hence, Method 2 proves to be more detailed not only in the variety of GO terms but also in the number of associated TF-DEGs. Regarding the genes left out by Method 1, we note that both TFAP2C and LHX2 were associated only with GO:00040029 regulation of gene expression, epigenetic, which results in being a high-level label. On the contrary, the new entry TRPS1 is associated with both GO:0002062 and GO:0032330, i.e., with chondrocyte differentiation. This means that Method 2 opted again in favor of a more CML-specific connotation.

## 5. Discussion

We analyzed the modulation of CML cell model K562 transcriptome following transfection with the tumor suppressor gene PTPRG and treatment with the tyrosine kinase inhibitor (TKI) Imatinib with the aim of identifying genes responding to the PTPRG modulation and/or treatments with Imatinib.

We developed two GSEA-based computational methods, Method 1 and Method 2, aimed at detecting all the CML-related differentially expressed transcription factors (TF-DEGs) and the biological processes involved. To summarize, the genes responsive to the treatments found by our methods are:

- Method 1:
  - Up-regulated TF-DEGs: MECP2, NR2E1, RARG;
  - Down-regulated TF-DEGs: ZBTB16, TFAP2C, SOX5, SMAD1, LHX2, IKZF3, IFI16, EPAS1, BATF3, BACH2;
- Method 2:
  - Up-regulated TF-DEGs: MECP2, NR2E1, RARG;
  - Down-regulated TF-DEGs: ZBTB16, TRPS1, SOX5, SMAD1, IKZF3, IFI16, EPAS1, BATF3, BACH2.

Method 1 was designed to take as input the whole list of DEGs stemming from DEA and afterwards select only the TF-DEGs. On the other hand, Method 2 was set to filter out only TF-DEGs, identifying a smaller gene universe than Method 1. Moreover, the two methods were split into two modalities to discern between up-regulated and down-regulated genes. We observed that Method 1 returned more GO labels than Method 2 in both modalities. However, this entailed different outcomes for up-regulated and down-regulated TF-DEGs, respectively. In fact, both the word clouds and the correlation analysis showed that for up-regulated TF-DEGs, Method 1 returned appropriate and specific GO labels, while Method 2 provided more general results. Nevertheless, the selections of CML-related TF-DEGs stemming from key term analysis identified the same list of genes for both methods. Hence, we could say that in this case, Method 1 appears to be more appropriate on the grounds that it identified more specific GO labels than Method 2. For down-regulated TF-DEGs, Method 1 provided more high-level biological insights at all stages (weighted word clouds, correlation analysis, and key term selection), while Method 2 showed more specific references to CML-related biological processes. However, the final lists of CML-related TF-DEGs differ for only a few genes (LHX2 only for Method 2, and TFAP2D and TRPS1 only for Method 1). In this case, Method 2 is to be preferred to Method 1.

It is worth underlining that the validity of the dataset used and the methods applied were validated in the cell model by the identification of a novel molecular pathway that has been suggested by the analysis of the data derived from the approach proposed. Based on the identification of RARG and CD36 regulation in PTPRG overexpressing K562, we reasoned that a cell differentiation pathway might be altered in this condition. Indeed, Walkley et al. [34] showed that RARγ null mice exhibit a considerable increase in granulocytes in the peripheral blood (PB) and in the bone marrow (BM) and spleen, developing a myeloproliferative-like syndrome and displaying a reduction in the megakaryocyte–erythroid progenitor fraction, thus altering homeostatic bone marrow erythropoiesis. We were able to confirm CD36, a Scavenger receptor expressed in myeloid cells [52], up-regulation at the protein level and found that this was associated with hemoglobin overexpression, both markers of erythromyeloid differentiation induced by the treatment with TKI Imatinib. These data further confirm the key role of PTPRG, a gene described as a key regulator of cell differentiation in normal and in CML cells, acting as tumor suppressor in this context, a role supported by several studies performed in patients [15,16,20,21,53]. Strategies aimed at restoring PTPRG expression are expected to benefit the course of the disease by improving drug efficacy or contrasting the emergence of BCR::ABL1 mutants.

In conclusion, the methods here presented offer a versatile exploratory computational approach to analyzing and extracting meaningful biological information. The study combines statistical tests for DEA and GSEA with human-curated contents (Gene Ontology), weighted word clouds, correlation analysis, and key term selection, originally born in different application domains (such as textual analysis). These methods could also potentially be very useful and expressive in the descriptive statistical analyses applied to gene biology.

Finally, we provide some future development of our analysis. The identification of genes responsive to pharmacological treatments is certainly not limited to the application of these exploratory methods focused mainly on the gene as a single entity and the quantitative characteristics of its activity (e.g., its expression level), but requires analyses relevant to the field of systems biology. Indeed, the past 20 years have seen a revolution in the volume and complexity of data generated in experiments and observations in the life sciences. With the increase in available data, the need for data management, integration, and analysis has become an increasingly important challenge. Biological knowledge is inherently complex and so cannot readily be integrated into existing databases of molecular data. For more than 20 years, ontologies have provided a means of unambiguously specifying biological knowledge—for example, about genes, anatomy, and phenotypes—in complex graph-based structures, which formally represent the concepts that are relevant in the domain and the relationships between them [54]. On the one hand, an ontology defines a vocabulary of terms to denote concepts and relationships that are familiar to the user. On the other hand, it extends the data with background knowledge, such as sub-class and sub-property axioms, axioms establishing which classes constitute the domain and range of properties, and axioms expressing the disjointedness between classes or properties.

The use of ontologies began in the biological sciences around 1998 with the development of the Gene Ontology [55,56], which systematically summarizes the current knowledge of gene products across a wide range of species. Since then, many other initiatives have given rise to the design and implementation of ontology-based data management systems (also known as “Virtual Knowledge Graphs” [57,58] in the biological domain). Since then, many other databases have been created to store biological information in ontological structures. We refer the reader to [56,59,60] for a comprehensive review of the most relevant existing ontologies in this field and their associated data sources.

Currently, the most prominent ontologies and ontology-based data management systems in the biology field store knowledge and data about the static structures of biological organisms, whereas the dynamic behaviours of biological processes have, for the past half-century, been captured in the mathematical language of physics-based simulation modelling [61]. To date, there have been only a few attempts to bridge the wealth of structural knowledge and the wealth of process knowledge, i.e., of the physico-chemical laws described by equations of dynamical models. D. Cook et al. [61] introduced the terms *bio-ontology* and *biosimulation* to indicate ontologies related to biological entities and simulation of physics-based mathematical models of biological systems dynamics.

Specifically, D. Cook and co-authors showed that the semantics of biosimulation models could be expressed in a formal ontology that describes the entities, the properties, and the physical laws that are encoded in the mathematical equations of a simulation model. They introduced the Ontology of Physics for Biology (OPB) [62,63] based on systems dynamics that makes explicit the biophysical semantics of physics-based biosimulation models. OPB can be used as a reference knowledge resource for annotating variables and equations of models and for deriving computable modeling code. Therefore, the future direction of this study is the development of a methodology to bridge this gap and link the semantics of biosimulation to the knowledge in structural bio-ontologies. A possible way to pursue this goal could be the analysis of gene networks resulting from the identification of TF-DEGs of interest. More specifically, we plan to choose TF-DEGs that seem to be involved in CML-related biological processes and expand the analysis on genes that interact with them. The types of relations between genes can be retrieved from various sources as partner or pathway databases. Here we relied on Pathway Commons, a pathway database that uses the Biological Pathway Exchange (BioPAX) [64] standard to represent data. It allows for investigating multiple biological concepts, such as biochemical reactions; gene regulatory networks; genetic interactions; proteins, small molecules, DNA, RNA, complexes, and their cellular locations; complex assembly and transport; post-translational protein modifications; citations; experimental evidence; and links to other databases, e.g., protein sequence annotation [65].

For our purposes, we focused on two types of gene–gene relationships involving TF-DEGs:

- Control of gene expression (one-way relationship): we analyzed all the genes in control or controlled by TF-DEGs in terms of expression levels.
- Interaction between genes (two-way relationship): we analyzed all the genes that chemically interact with TF-DEGs.

Since the analysis on up-regulated genes returned the same set of relevant TF-DEGs, we focused only on down-regulated genes. Figures S2–S5 in Supplementary Materials show the analysis results for Method 1 and Method 2 as gene networks. In conclusion, we plan to investigate the biological and chemical relations between the genes represented in the networks to enrich the exploratory methods hereby defined with additional information about the network dynamics. The construction of the equations for the dynamics of the gene networks of interest involves calibrating the model as the next step. In possession only of static data, such as those used in this study, this phase will require the development of efficient sensitivity analysis techniques, given the large number of genes potentially involved and the expected non-linear dynamics. In this regard, we plan to refine the numerical techniques for parameter sensitivity analysis, inference, and the dynamic simulation developed in [66,67,68].

**Figure 16:**
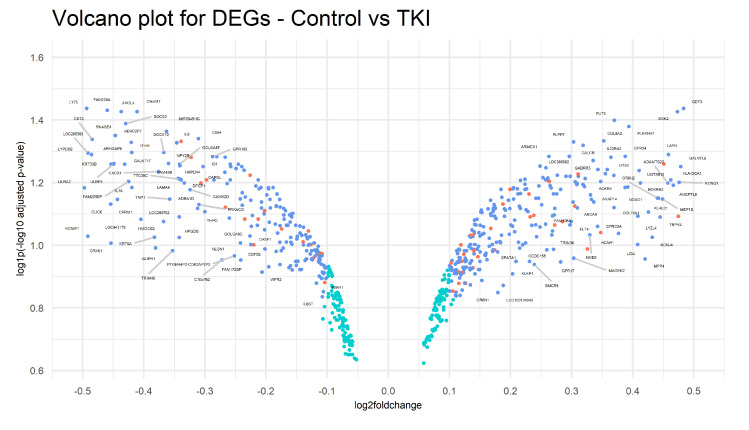
Volcano plot plot for DEGs. Genes are represented as scattered points: the *x*-axis is the log2FoldChange, and the *y*-axis shows the log1p(−log10 adjusted *p*-value). Both red and blue dots represent genes that were identified as significantly differentially expressed (adjusted *p*-value < 0.05) with |log2FoldChange| > 0.1. Specifically, red dots refer to transcription factors.

**Figure 17:**
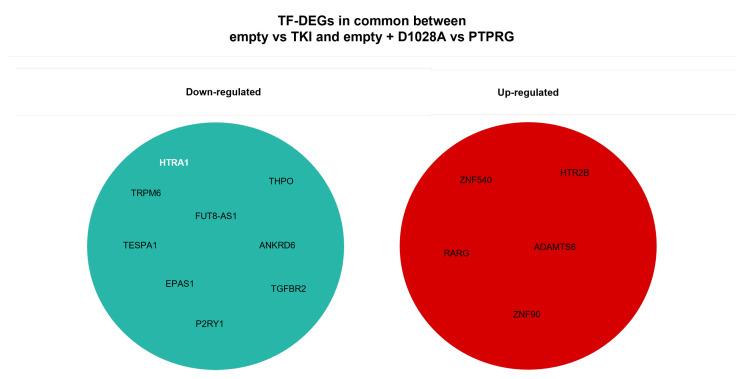
TF-DEGs in common between empty vs TKI and empty + D1028A vs PTPRG. Down-regulated genes are represented in the first set, whilst up-regulated ones are shown in the second set. Moreover, TF-DEGs are written in bold white, and DEGs are reported in black.

Another future research line to be further explored is the identification and the analysis of the DEGs responsive to both the case study under examination and known pharmacological treatments with TKI. In this direction, we preliminarily performed DEA to detect DEGs between two groups: control considered as the untreated group (two replicates) and the treatment group referred to as TKI-expressing samples (two replicates). In order to discard background noise, only genes with an intra-group standard deviation <0.3 and distance between the group means >0.5 were considered. Differential expression analysis was conducted on log2-transformed gene expressions using the Bioconductor/R package limma (Version 3.12). Both the empirical Bayes correction on the variances and the multi-testing Bonferroni–Hochberg correction on *p*-values were selected. Therefore, genes with an adjusted *p*-value < 0.05 and |log2FoldChange|> 0.1 were considered to be differentially expressed. Based on these criteria, 568 genes have been selected as DEGs: 310 were up-regulated, and 258 were down-regulated. Among them, we have identified 61 transcription factors, of which 25 are down-regulated, and 36 are up-regulated (see Figure 16). The top five down-regulated TFs are GATA3, RUNX3, HES1, TBX4, and FOSL1, while the top five up-regulated are NPAS4, FOXN4, HOXA2, PURG, and ZNF540. Then, we selected only the DEGs that occurred in both selections stemming from DEA. The results are represented in Figure 17 and are split between up-regulated and down-regulated genes.

## 6. Conclusions

In this study, we identified molecular pathways modulated by the tumor suppressor gene PTPRG and focused on transcription factors known to act as master genes controlling a high number of downstream effectors. Future avenues might involve the identification of specific genes regulated by them and investigating the biological and chemical relations between the genes represented in the networks to enrich the exploratory methods hereby defined with additional information about the network dynamics. The methods here presented offer a versatile exploratory computational approach to analyze and extract meaningful biological information. The study combines statistical tests for DEA and GSEA with human-curated contents (Gene Ontology), weighted word clouds, correlation analysis, and key term selection, originally born in different application domains (such as textual analysis). Of note, we have validated the microarray data using a group of differentially expressed genes and identified a cell differentiation program activated by the TSG PTPRG, leading to a higher propensity of the blasts to differentiate toward a more mature phenotype, a condition that is further enhanced by TKI treatment. These data further support the relevance of the re-expression of PTPRG in the context of CML, suggesting it as a relevant therapeutic target. These methods also could potentially be very useful and expressive in the descriptive statistical analyses applied to gene biology.

## Figures and Tables

**Table 1:** Sequences of gene-specific primers used in this study.

Gene	Forward 5${}^{\prime }$–3${}^{\prime }$	Reverse 5${}^{\prime }$–3${}^{\prime }$
MECP2	CGTGAAGGAGTCTTCTATCCGA	GCTTCACCACTTCCTTGACC
TFAP2C	ATTCGCAAAGGTCCCATTTCC	GGCATTTAAGCATTCAGGTGG
RARG	GCAAGTATACCACGAACTCCAG	ACGCAGCATCAGGATATCTAGG
TRPS1	CAAACAAGAAGCAAATCACCTG	GTGTGCTCTCCTGTAGTGTC
SMAD1	TCCTTCCAACAATAAGAACCGT	CTACTGTCACTAAGGCATTCG
CD36	TTTGGCTTAATGAGACTGGGAC	ACAAACATCACCACACCAACAC

**Table 2:** GO terms of the biological processes selected by up-regulated TF-DEGs returned by Method 1. We note that the majority of GO labels are associated with the terms chromatin and acetylation, while there is only one GO label (GO:0045637) directly correlated to CML.

Chunk	References	GO ID
chromatin	[37]	GO:0016569, GO:0034401, GO:0097549,
		GO:1905269, GO:0006342
acetylation	[38]	GO:0006473, GO:0006475, GO:0018393,
		GO:0016573, GO:0018394
acylation	[39]	GO:0043543
amino acid	[40]	GO:0018193
cell growth		GO:0016049
myeloid cell	[41]	GO:0045637
differentiation		
angiogenesis	[42]	GO:0090049

**Table 3:** GO terms of the biological processes selected by up-regulated TF-DEGs returned by Method 2. We note that as opposed to Method 1, the selection comprises just a few terms. Furthermore, only one term (GO:0045637) appears to be strictly correlated to CML.

Process	References	GO ID
regulation of myeloid	[41]	GO:0045637
cell differentiation		
regulation of blood vessel	[43]	GO:0043535
endothelial cell migration		

**Table 4:** GO terms of the biological processes selected by Method 1 for down-regulated genes. We note that most of the GO labels are associated with the term differentiation. Moreover, the other terms comprised in the selection also appear to be clearly correlated with CML.

Process	References	GO ID
growth		GO:00071559, GO:0071560
differentiation	[41]	GO:0046637, GO:0006475, GO:0018393,
		GO:0016573, GO:0046632
immune		GO:0002376, GO:0002253
kinase	[41]	GO:0007178
epigenetic	[44]	GO:0040029
endopeptidase	[45]	GO:2000117
cell population		GO:0045637

**Table 5:** GO terms of the biological processes selected by Method 2. We note that the terms differentiation, immune, and leukocyte are the most significant as far as number of associated GO labels. Moreover, also the other terms comprised in the selection appear to be strictly correlated to CML. In this case, the correlation is clearly higher than the one returned by Method 1. This is due to the fact that the key terms comprised in the selection refer to more specific biological processes involved in CML.

Process	References	GO ID
leukocyte	[46]	GO:0002521, GO:1902105, GO:0045321,
		GO:0002573
immune		GO:0002376, GO:0006955, GO:0002520,
		GO:0002550
chondrocyte	[47]	GO:0002062, GO:0032330
p53	[48]	GO:0072331
myeloid	[41]	GO:0030099, GO:0002573
growth		GO:0071560, GO:0071559
differentiation	[41]	GO:0002521, GO:1902105, GO:0030099,
		GO:0002573, GO:0032330
hemopoiesis	[49]	GO:0030097
hematopoietic	[49]	GO:0048534
cytokine	[50]	GO:0001816, GO:0001817
phosphorylation	[50]	GO:0006468
stem	[51]	GO:0098722, GO:0008356

## Data Availability

The gene dataset and the software scripts used for the analysis of data can be found at the following GitLab link: https://gitlab.inf.unibz.it/Paola.Lecca/chronic-myeloid-leukemia-genes (accessed on 9 August 2022).

## Supplementary Materials

The following supporting information can be downloaded at: https://www.mdpi.com/article/10.3390/ijms23179899/s1.

## Abbreviations

DEG	Differential Expression Gene
TF-DEG	Transcription Factor Differential Expressed Gene
DEA	Differential Expression Analysis
GSEA	Gene Set Enrichment Analysis
GO	Gene Ontology
OPB	Ontology of Physics for Biology
CML	Chronic Myeloid Leukemia
IMA	Imatinib
Ct	Cycle threshold
RT-PCR	Real Time Polymerase Chain reaction
TSG	Tumor Suppressor Gene
