# Supplementary material for: Gene Expression Landscape of Chronic Myeloid Leukemia K562 Cells Overexpressing the Tumor Suppressor Gene PTPRG
Source: Int J Mol Sci. 2022 Aug 31;23(17):9899. doi: 10.3390/ijms23179899 (PMC9456469; doi:10.3390/ijms23179899)
Supplement: Supplementary file 1 [file ijms-23-09899-s001.zip › ijms-1819560-supplementary.pdf]

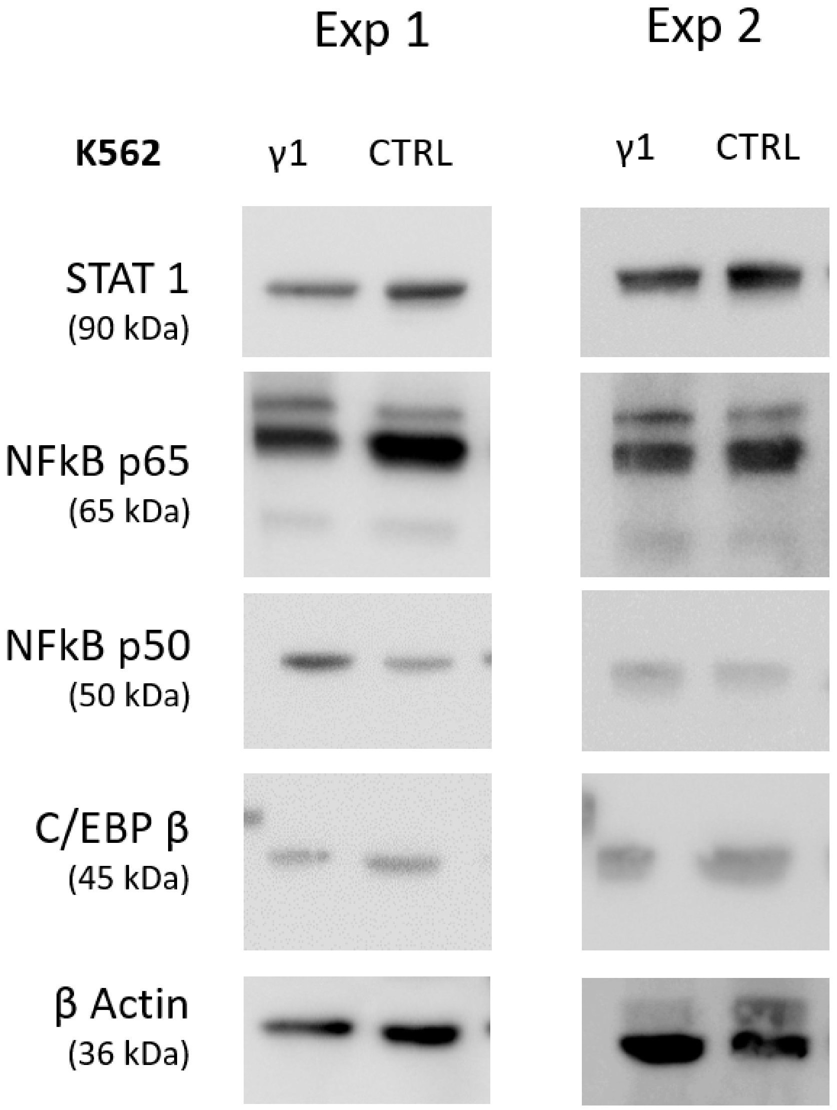

Figure S1. Western blot analysis of the expression levels of the transcription factors STAT1, NFκB p50, C/EBP β and NFκB p65. β actin was used as loading control. 20 μg total protein was loaded for each sample.

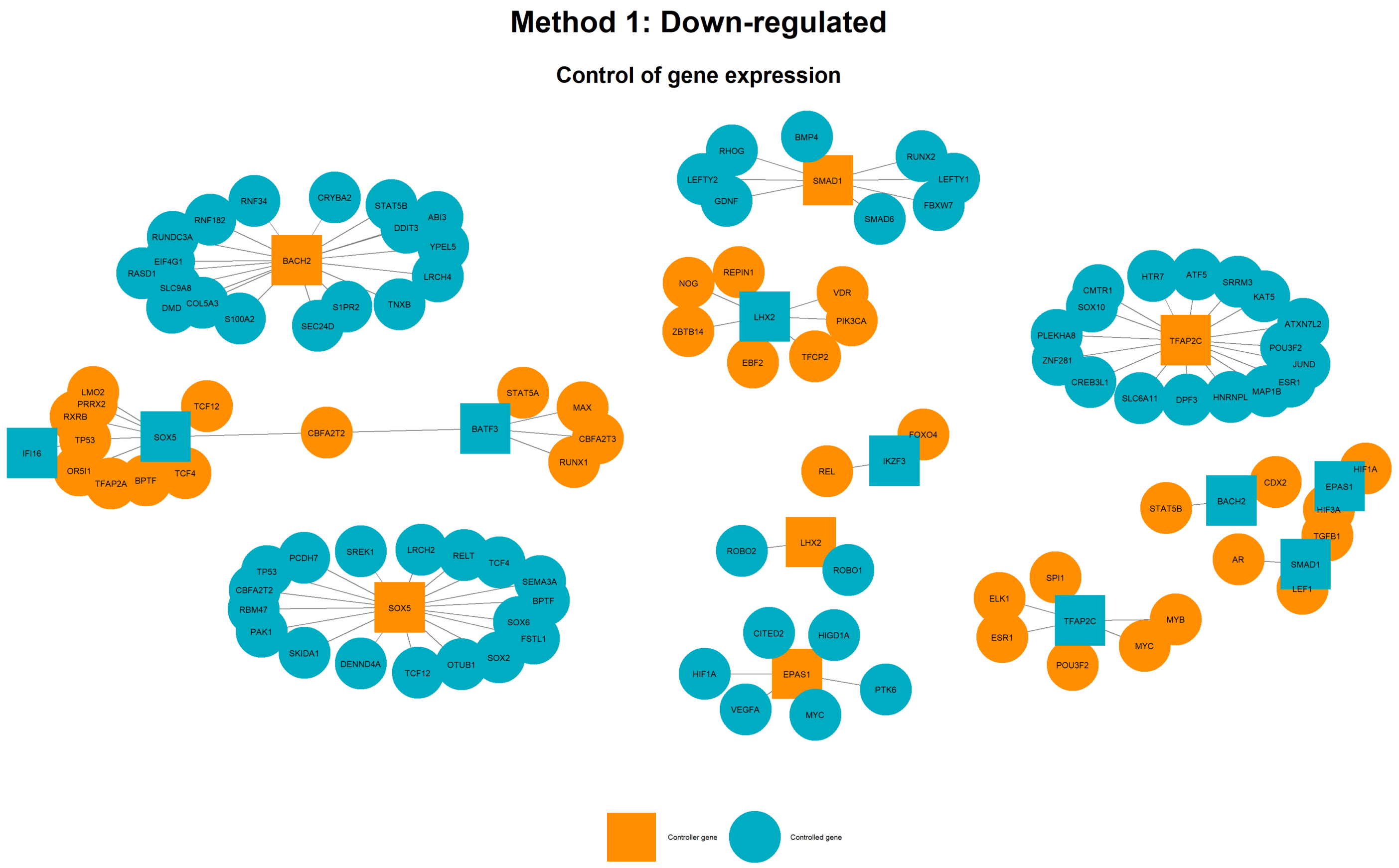

Figure S2. Method 1: genes correlated to TF-DEGs by the function "control of gene expression". TF-DEGs are square-shaped while gene partners are represented by circles. Orange labels are referred to genes that act as controllers while cyan is assigned to genes that are controlled by others. We note that the nets originating from IFI16, SOX5 and BATF3 are interconnected through some common genes.

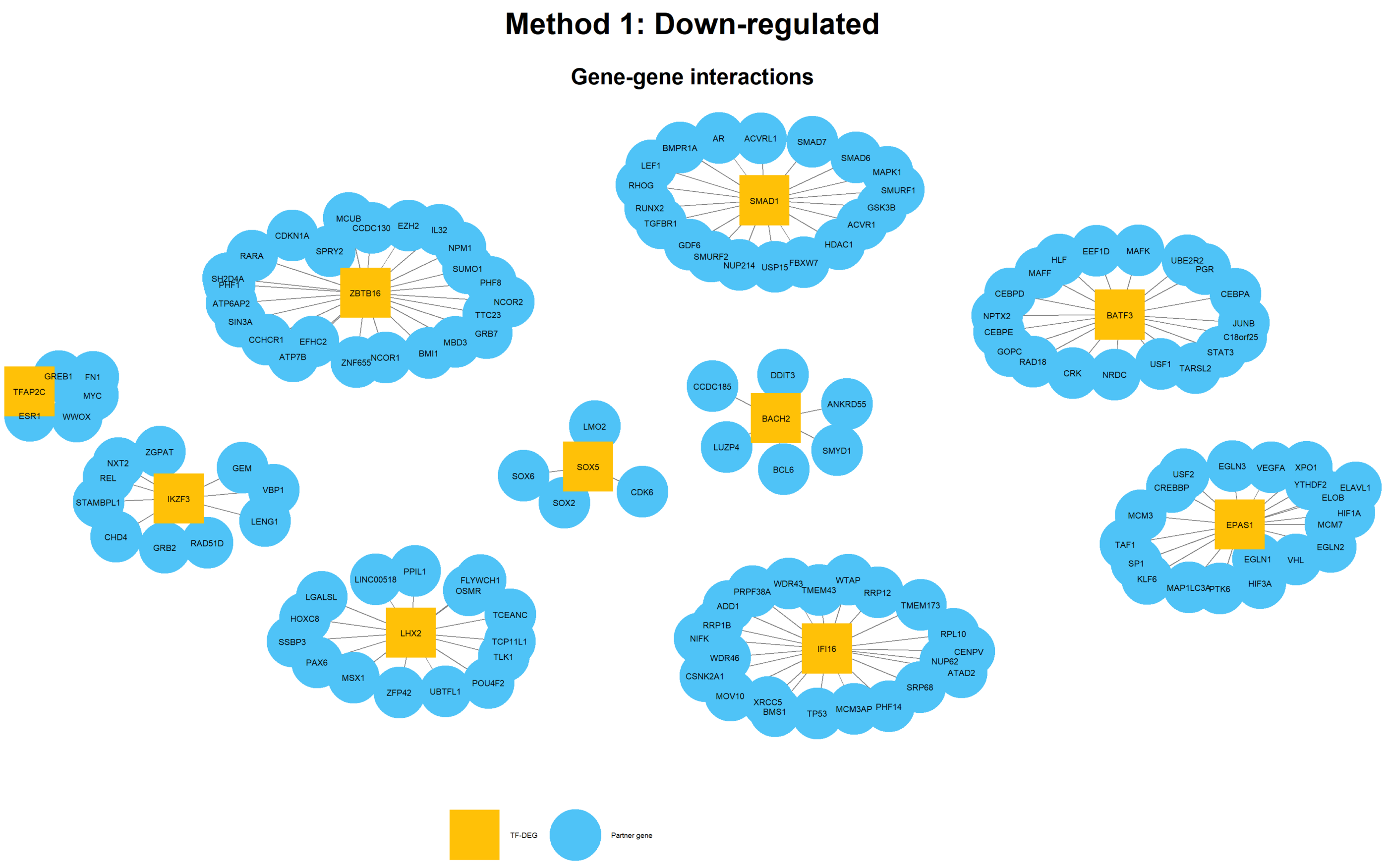

Figure S3. Method 1: genes that interact with TF-DEGs. TF-DEGs are square-shaped while gene partners are represented by circles. Yellow labels are referred to TF-DEGs while light blue is assigned to their partners. We note that there are no interconnected nets.

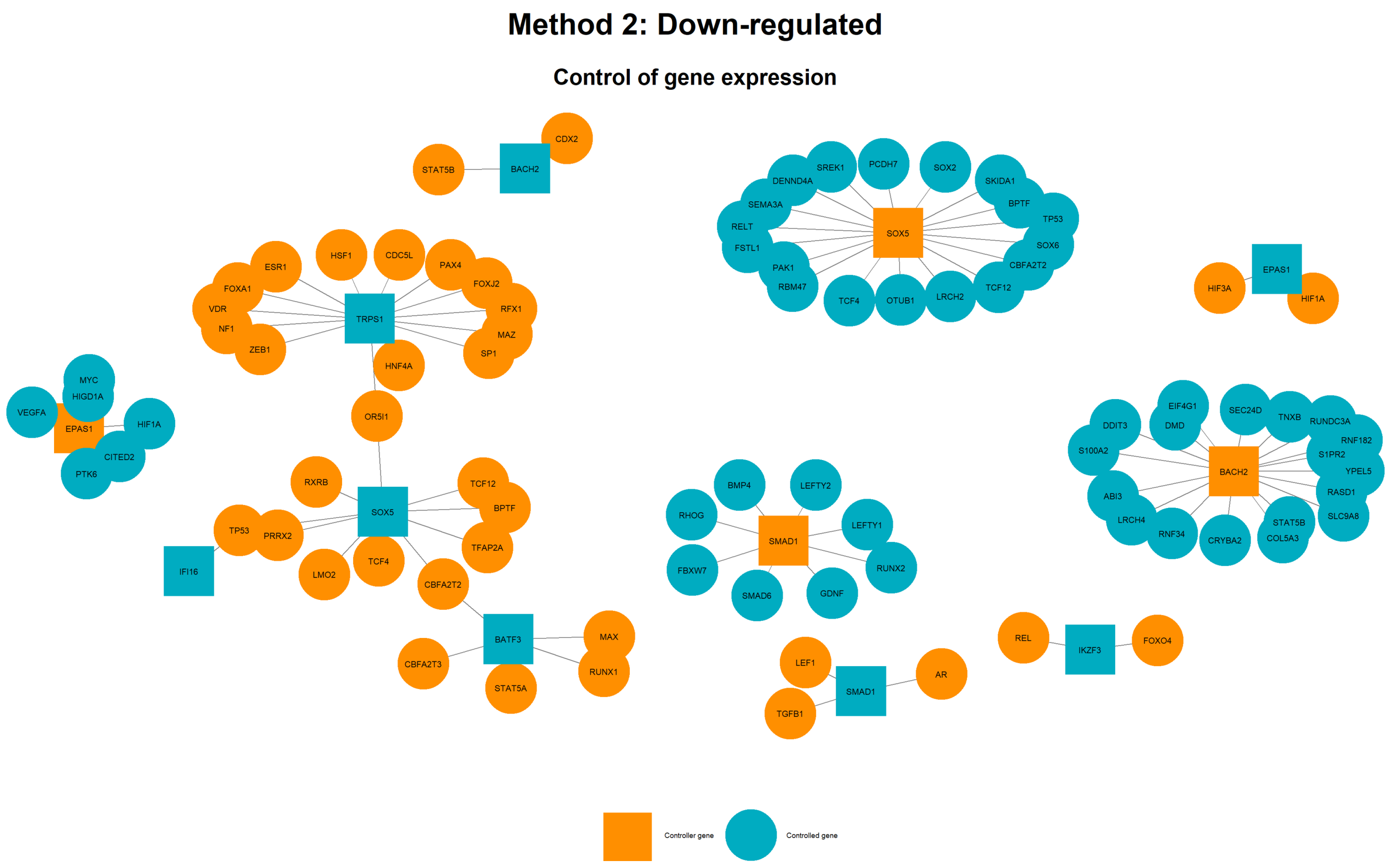

Figure S4. Method 2: genes associated to TF-DEGs by a relationship of the kind "control of gene expression The plot shows all the genes associated to TF-DEGs by a relationship of the kind "control of gene expression". TF-DEGs are square-shaped while gene partners are represented by circles. Orange labels are referred to genes that act as controllers while cyan is assigned to those genes that are controlled by others. We note that the nets originating from TRPS1, SOX5, IFI16 and BATF3 are interconnected through some common genes.

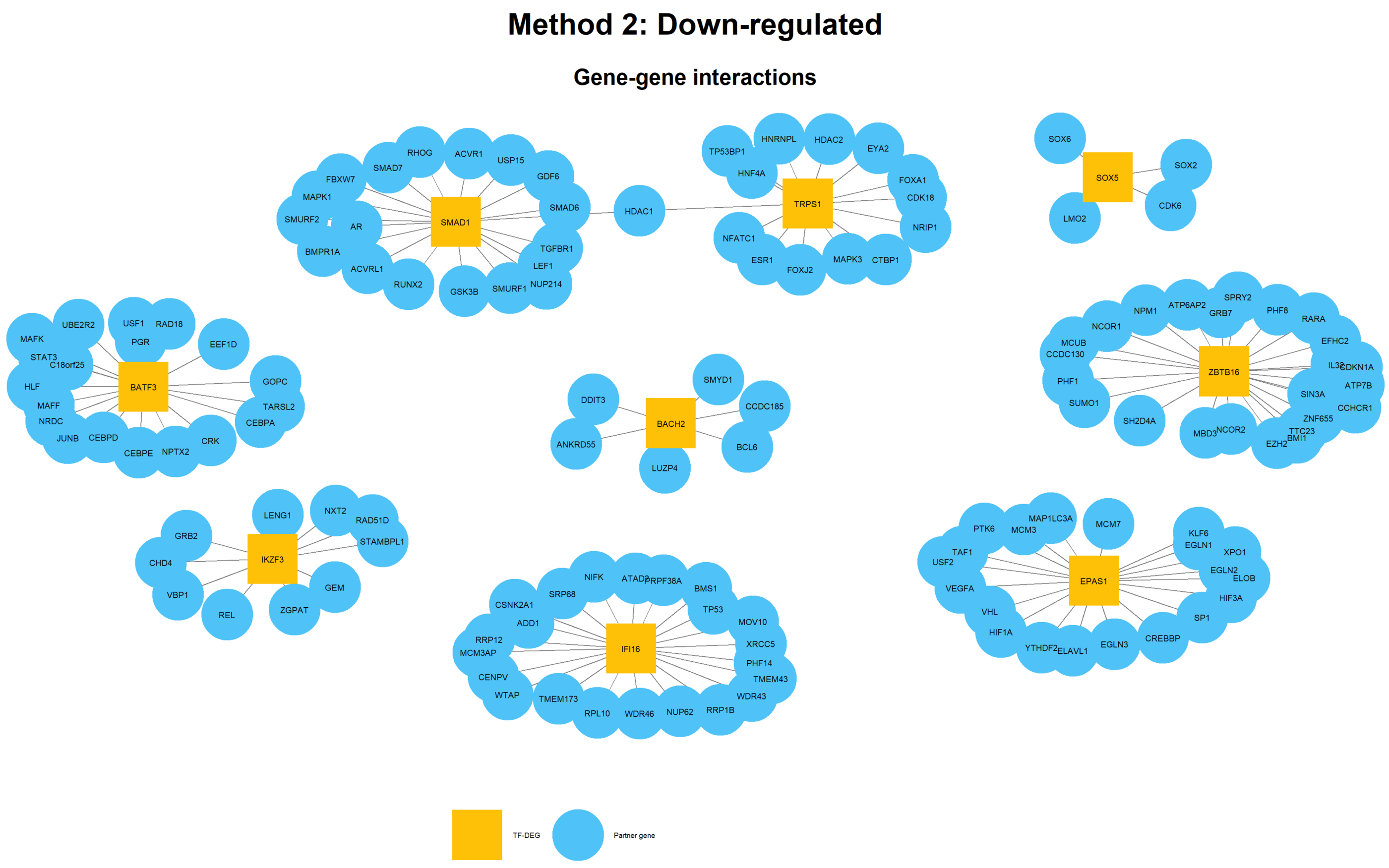

Figure S5. Method 2: genes that interact with TF-DEGs. TF-DEGs are square-shaped while gene partners are represented by circles. Yellow labels are referred to TF-DEGs while light blue is assigned to their partners. We note that the nets originating from SMAD1 and TRPS1 are interconnected through gene HDAC1.
